# Our Added Years

**Published:** 1896-08

**Authors:** 


### OUR ADDED YEARS.

With this number of the Journal closes the second year since the resuscitation with the September issue of 1894. For the kindly support of the profession in maintenance, for the wealth of material contributed by our friends, for the kind words of encouragement and the warm words of praise received, the editorial management are deeply thankful and grateful to all. While new duties, broader plans, and many changes in the business management and direction have from time to time compelled more rigid rules in regard to subscriptions, there has been but one purpose in view in so doing, viz., a general rule, applied to all, and to make the JOURNAL better and stronger in every department. Every dollar received, and much more besides, has been used in strengthening the JOURNAL, that it might thereby better fulfil its mission for the manifold interests of the profession. In the future, as in the past, our watchword will be to "Lead Veterinary Journalism in America." Faithful to the interests of the profession, zealous for its welfare, and earnest in the maintenance of a high ethical standard, the JOURNAL will be independent in all things, neutral in nothing. Believing that such a journal is a factor in the work of the veterinarian, we ask at the hands of the profession a stronger support in the future, that we may better discharge the exacting duties of editorial direction and management.

